# Morphology Controlled Deposition of Vanadium Oxide (VO_x_) Nanoparticles on the Surface of Highly Reduced Graphene Oxide for the Photocatalytic Degradation of Hazardous Organic Dyes

**DOI:** 10.3390/ma16186340

**Published:** 2023-09-21

**Authors:** Mohammed Rafi Shaik, Fatimah N. Aldhuwayhi, Amal Mohammed Al-Mohaimeed, Mohammad Rafe Hatshan, Mufsir Kuniyil, Syed Farooq Adil, Mujeeb Khan

**Affiliations:** Department of Chemistry, College of Science, King Saud University, P.O. Box 2455, Riyadh 11451, Saudi Arabia; mrshaik@ksu.edu.sa (M.R.S.); 441203388@student.ksu.edu.sa (F.N.A.); muhemeed@ksu.edu.sa (A.M.A.-M.); mhatshan@ksu.edu.sa (M.R.H.); mkuniyil@ksu.edu.sa (M.K.); sfadil@ksu.edu.sa (S.F.A.)

**Keywords:** vanadium oxide, highly reduced graphene, organic dyes, photocatalytic, degradation

## Abstract

Semiconducting nanomaterials based heterogeneous photocatalysis represent a low-cost, versatile technique for environmental remediation, including pollution mitigation, energy management and other environmental aspects. Herein, we demonstrate the syntheses of various heterogeneous photocatalysts based on highly reduced graphene oxide (HRG) and vanadium oxide (VO_x_)-based nanocomposites (HRG–VO_x_). Different shapes (rod, sheet and urchin forms) of VO_x_ nanoparticles were successfully fabricated on the surface of HRG under solvo-/hydrothermal conditions by varying the amount of water and ethanol. The high concentration of water in the mixture resulted in the formation of rod-shaped VO_x_ nanoparticles, whereas increasing the amount of ethanol led to the production of VO_x_ sheets. The solvothermal condition using pure ethanol as solvent produced VO_x_ nano-urchins on the surface of HRG. The as-prepared hybrid materials were characterized using various spectroscopic and microscopic techniques, including X-ray diffraction, UV–vis, FTIR, SEM and TEM analyses. The photocatalytic activities of different HRG–VO_x_ nanocomposites were investigated for the photodegradation of methylene blue (MB) and methyl orange (MO). The experimental data revealed that all HRG–VO_x_ composite-based photocatalysts demonstrated excellent performance toward the photocatalytic degradation of the organic dyes. Among all photocatalysts studied, the HRG–VO_x_ nanocomposite consisting of urchin-shaped VO_x_ nanoparticles (HRG–VO_x_-U) demonstrated superior photocatalytic properties towards the degradation of dyes.

## 1. Introduction

Semiconducting nanomaterial-based heterogeneous photocatalysis is a low-cost, versatile technique for environmental remediation, including pollution mitigation, energy management and environmental aspects [1,2]. Since the pioneering work of Fujishima and Honda in 1970s, this method has been successfully utilized for the removal of a variety of biological, organic and inorganic pollutants from water and air; for chemical syntheses; and also for the production of clean energy [3,4]. Typically, the process of photocatalysis involve two steps: the generation of excited electron–hole charge carriers through the absorption of light and the subsequent use of photo-excited charge carriers as active catalytic centers to initiate oxidation/reduction reactions [5,6]. However, the advancement in the field of photocatalysis is often hampered by the inefficient usage of visible light, low quantum efficiency and the instability of the photocatalytic materials [7]. Therefore, the development of improved, effective solar absorption and chemically stable photocatalysts have remained an enduring topic in the field of photocatalysis [8].

Usually, in heterogeneous photocatalysis involving advance oxidation processes (AOPs), metal oxide-based semiconductors like TiO_2_, ZnO, SnO_2_, V_2_O_5_ and CeO_2_, etc., are preferred, due to their low costs, unique electronic structure, efficient light absorption capacities and fast charge transport properties [9,10]. But the majority of metal oxides-based nanomaterials suffer from low efficiency under visible light (between 400 to 800 nm), limited chemical stability under experimental conditions and a high recombination rate of photogenerated electron–hole pairs and environmental compatibility [11]. To overcome these issues, the band gap of transitional metal oxides is typically altered via doping with other materials, including metallic and non-metallic atoms/particles, which ultimately enhance the light absorption capacity of the resulting composites [12,13]. Moreover, customized transition metal oxides also demonstrate great chemical stability, and because of their abundance in earth, they exhibit decent environmental compatibility [14]. In addition, the light adsorption capacity of metal oxides can also be enhanced by extending the electron/hole recombination lifetime [15].

This is typically achieved by supporting the transition metal oxides over efficient support materials consisting of a large surface area for high adsorption capacity, excellent conductivity for fast charge transfer and so on [16]. In this context, carbonaceous materials, like carbon nanotubes and graphene, are highly preferred, due to their desirable physicochemical properties, including high surface area and electrical conductivity [17]. Therefore, the combination of metal oxides and 2D materials, including graphene, offers excellent benefits in the field of photocatalysis, due to the coordination and synergic effect of the components involved in the resulting nanocomposites [18]. Particularly, graphene derivatives, including pristine graphene (single-layer), graphene oxide (GO) and highly reduced graphene oxide (HRG), offer extraordinary properties as supports for various materials. Although pristine graphene is highly effective, due to its extremely high specific surface area and strong conjugation properties (defect less), it is usually not preferred in catalysis, which often requires large quantities of materials [19]. Moreover, defects play a crucial role in catalysis, as they offer plenty of active sites for the proper adsorption of materials on the surface of catalysts. And thus, in comparison to the less pristine, defective graphene, HRG with plenty of defects is a suitable support for different types of active catalysts; moreover, HRG can also be prepared in large quantities using a variety of sequential oxidation–reduction approaches [20].

Among diverse metal oxides, vanadium oxides have attracted considerable interest from researchers due to their promising properties and varying oxidation states; at least 15 different phases caused by variation in the oxidation states of vanadium, ranging from V^5+^ to V^0+^, have been reported [21,22]. Vanadium belongs to the list of most abundant transition metals, and its oxide counterparts are highly crystalline in nature with different oxygen coordination, which leads to the formation of diverse phases, including octahedral, pentagonal bipyramids and so on [23]. The diverse oxidation states of cations have a significant impact on the physicochemical properties of the vanadium oxides with different phases. For instance, the oxides of vanadium exhibit superb intercalation characteristics for host–guest molecules or ions, excellent catalytic activities, strong electron–electron correlations and outstanding phase transitions properties [24]. Of the various phases, VO_2_ has a fascinating narrow band gap (i.e., 0.7 eV) consisting of V with a oxidation state of +4 [25]. It is capable of switching between a *monoclinic* and a *tetragonal* phase at facile conditions (∼68 °C), which corresponds to a semiconductor-to-metal transition, respectively [26]. Due to this, VO_2_ is considered a promising material for a variety of applications in electrochromic, fast-switching and other electronic applications [27]. The narrow band gap of VO_2_ has largely inhibited its widespread photocatalytic applications. On the other hand, the possibility of the crystallization of VO_2_ nanoparticles in different sizes, shapes and morphologies has broadened the chances of its photocatalytic applications [28]. In addition, different shapes of VO_2_ nanoparticles can be beneficial in enhancing the photocatalytic properties of the resulting catalyst, due to the change in surface properties, crystallographic planes, packing and density [29].

Therefore, it is of interest to study the shape-dependent photocatalytic properties of VO_2_ nanoparticles [30]. The effective adjustment of kinetics and the thermodynamics of crystal growth are crucial parameters for controlling the final morphology of nanocrystals [31]. This is typically achieved by altering the reaction conditions and the utilization of surfactants, foreign particles or molecules, among others [32]. However, the strategies of generating varied shapes of metal oxide nanoparticles on different support materials are relatively less reported when compared to the direct solution-based fabrication of metal and metal oxide nanoparticles in varied morphologies [33]. Indeed, to the best of our knowledge, the preparation of different shapes of VO_x_ nanoparticles on the surface of highly reduced graphene oxide (HRG) for photocatalytic applications has not been reported so far.

Thus, in this study, we report the preparation of a variety of HRG–VO_x_ nanocomposites, wherein the different shapes of VO_x_ nanoparticles (NPs) were deposited on the surface of HRG in a facile solvo-/hydrothermal reaction. In the presence of HRG, the shapes of the VO_x_ NPs were controlled by adjusting the ratio between water and ethanol. The morphologies of NPs on the surface of HRG were determined via scanning electron microscopy (SEM) and high-resolution transmission electron microscopy (HR-TEM). The composition and structure of the resulting nanocomposites were investigated using XRD, UV analysis, FTIR and XPS. Furthermore, the photocatalytic properties of as-prepared nanocomposites were evaluated for the degradation of methylene blue and methyl orange under UV light irradiation.

## 2. Materials and Methods

### 2.1. Materials

Graphite powder was purchased from Alfa Aesar Chemical Reagent Co. (Ward Hill, MA, USA). Vanadyl acetylacetonate (98%, VO(acac)_2_), pluronic F-127, ethanol (p.a.), methylene blue and methyl orange were purchased from Sigma Aldrich. All chemicals were of analytical grade and were used as received without further treatment.

### 2.2. Synthesis of HRG–VO_x_ Nanocomposites

For this purpose, highly reduced graphene oxide (HRG) was prepared according to our previously published study [34]. Briefly, the ratio between the ethanol and water was adjusted for the preparation three different HRG–VO_x_ nanocomposites with varied morphologies of VO_x_ on the surface of HRG. These samples were designated as HRG–VO_x_ nanorods (HRG–VO_x_-R), HRG–VO_x_ nanosheets (HRG–VO_x_-S) and HRG–VO_x_ urchins (HRG–VO_x_-U). Notably, to prepare all the nanocomposites, equivalent weights of HRG and vanadium precursors were used. For example, to synthesize HRG–VO_x_-R, 150 mg of Pluronic F-127 was dissolved in 5 mL of ethanol, to which 140 mg of VO(acac)_2_ and 140 mg of HRG were added, and the mixture was stirred at room temperature for 3 h. Subsequently, the resulting mixture was transferred to a 50 mL Teflon-lined autoclave to which another 20 mL of water was added. The autoclave was tightly sealed and kept in a furnace at 453 K (180 °C) for 24 h. After this, the product was isolated via centrifugation (15 min, 9000 rpm) and the remaining solid residue was washed with 15 mL ethanol, then dried in a vacuum oven at 313 K (40 °C) for 12 h. The other two composites were also prepared in a similar fashion, except for the change in the amount of ethanol which was used to dissolve the Pluronic acid. For example, in the case of HRG–VO_x_-S, instead of 5 mL of ethanol, 150 mg of Pluronic F127 was dissolved in 24.7 mL of ethanol, and then 140 mg of VO(acac)_2_ and 140 mg of HRG were added and the solution was stirred for same length of time (4 h). After this, 0.3 mL of water was added and the solution was transferred into a 50 mL Teflon-lined autoclave (the additional 20 mL of water was not added to the autoclave in this case) and kept at 453 K (180 °C) for 24 h, whereas to prepare the HRG–VO_x_ urchin, i.e., HRG–VO_x_-U, 150 mg of Pluronic F-127 solution was dissolved in 25 mL of ethanol, then 140 mg of VO(acac)_2_ and 140 mg of HRG were added and the solution was stirred for 4 h. Subsequently, the solution was transferred into a 50 mL Teflon-lined autoclave and heated to 453 K (180 °C) for 24 h. For all the samples, the workup procedures, such as washing and drying, remained the same.

### 2.3. Photocatalytic Activity Studies

To test the photocatalytic dye degradation properties of all the nanocomposites, methylene blue (MB) and methyl orange (MO) were used as model compounds. For this purpose, the photocatalytic degradation reactions were performed according to our previously published study, and all three nanocomposites, namely HRG–VO_x_-R, HRG–VO_x_-S and HRG–VO_x_-U, were applied as photocatalysts [35]. Briefly, 1 mL of 0.01 M NaBH_4_ solution was added to 30 mL of 10^−3^ M MB and/or MO solutions under constant stirring. Shortly after this (~5 min), 100 mg of the photocatalyst was added, i.e., in this case, HRG–VO_x_-U. Prior to irradiation, the samples were kept in the dark for several hours to maintain dark absorption balance. Subsequently, the resulting mixture was kept under irradiation from a 24 W Philips light-emitting diode (LED) lamp (λ > 400 nm, 440 nm to around 700 nm), with a distance of ∼10 cm from the reaction valve. The reaction mixture was stirred vigorously until the original color disappeared, which indicated the completion of the reaction. The kinetics of the reaction was performed by frequently withdrawing a small amount of the sample and analyzing it using UV–vis spectroscopy, and the best catalyst was selected (HRG–VO_x_-U) to test the stability of the photocatalyst. And to do this, after the reaction, the same catalyst was isolated, washed and used for a separate reaction, and the procedure was repeated at least three times.

### 2.4. Characterization

The identities of the obtained photocatalysts were confirmed through a number of characterization techniques. Powder XRD patterns were recorded using a Bruker diffractometer (Cu Kα (λ = 1.5406 Å) X-ray source) (D2-Phaser, Bruker, Mannheim, Germany). SEM, TEM, and energy-dispersive X-ray (EDX) spectroscopy were performed on a Jeol, JED-2200 series (Akishima, Tokyo 196-8558, Japan) and a Jeol TEM model JEM-1011 (Japan) at 100 keV, respectively. XPS spectra were measured on a PHI 5600 Multi-Technique XPS (Physical Electronics, Lake Drive East, Chanhassen, MN, USA) using monochromatized Al Ka at 1486.6 eV. Peak fitting was performed using CASA XPS Version 2.3.14 software, Wilmslow, Cheshire, UK. FT-IR analysis was performed for the identification of functional groups on a PerkinElmer, 1000, FT-IR spectrometer, Waltham, MA, USA. The optical study and photocatalytic activity were studied with a Lambda 35 UV–Vis spectrophotometer (PerkinElmer, Waltham, MA, USA) in quartz cuvettes (with a path length of 1 cm) using distilled water as the reference solvent.

## 3. Results and Discussions

**Figure 1:**
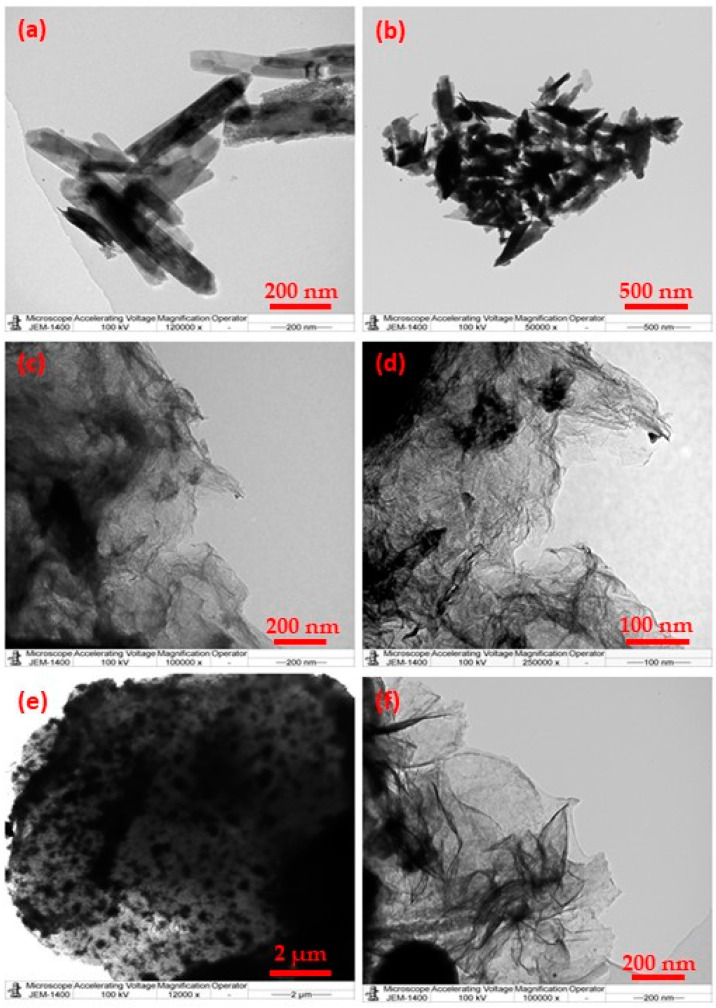
TEM images of HRG–VO_x_ nanocomposites consisting of different shapes of VO_x_ nanoparticles on the surface of graphene: (**a**,**b**) VO_x_ nanorods on HRG (HRG–VO_x_-R), (**c**,**d**) VO_x_ nanosheets on HRG (HRG–VO_x_-S) and (**e**,**f**) VO_x_ urchins on HRG (HRG–VO_x_-U).

The surfaces of photocatalysts play a crucial role in heterogeneous photocatalysis, as most of the photocatalytic reactions typically occur at the interface of the photocatalysts and liquid and/or gaseous reactants [36]. In this case, both the surface area and the surface atomic states are important and depend largely on morphology. Thus, the morphologies of the photocatalysts, which ultimately influence their surface areas, can potentially influence the resulting photocatalytic properties of the materials [37]. Typically, the size- and shape-controlled preparation of nanomaterials in solutions can be achieved through the precise adjustment of reaction parameters, including the temperature of the reaction and the pH, type and amount of solvent etc. [38]. In this study, we have fabricated highly reduced graphene oxide (HRG) and vanadium oxide (VO_x_) nanoparticle-based nanocomposites under solvo-/hydrothermal conditions by controlling the amount of water and ethanol. Three different HRG–VO_x_ nanocomposites were prepared, which consist of different shapes of VO_x_ nanoparticles, such as rods (HRG–VO_x_-R), sheets (HRG–VO_x_-S) and urchins (HRG–VO_x_-U). During the reactions, only the ratio of water and ethanol was varied, whereas the other reaction parameters like time, temperature, and the concentration of precursors were kept unchanged. The formation of different shapes of VO_x_ NPs on the surface of HRG was initially monitored via TEM analysis. During preparation, when varying the amount of water and ethanol, varied morphologies of VO_x_ NPs were formed. For instance, a high content of water in the reaction mixture, i.e., 20 mL of water and 5 mL of ethanol, yielded the formation of nanorods (cf. Figure 1a,b), while a higher amount of ethanol together with a small quantity of water led to the formation of sheets on the surface of HRG; in this case, 24.7 mL of ethanol and 0.3 mL of water were used (cf. Figure 1c,d). On the other hand, in the absence of water, pure ethanol alone generated urchin-like VO_x_ NPs with visible spikes on their surfaces (cf. Figure 1e,f).

**Figure 2:**
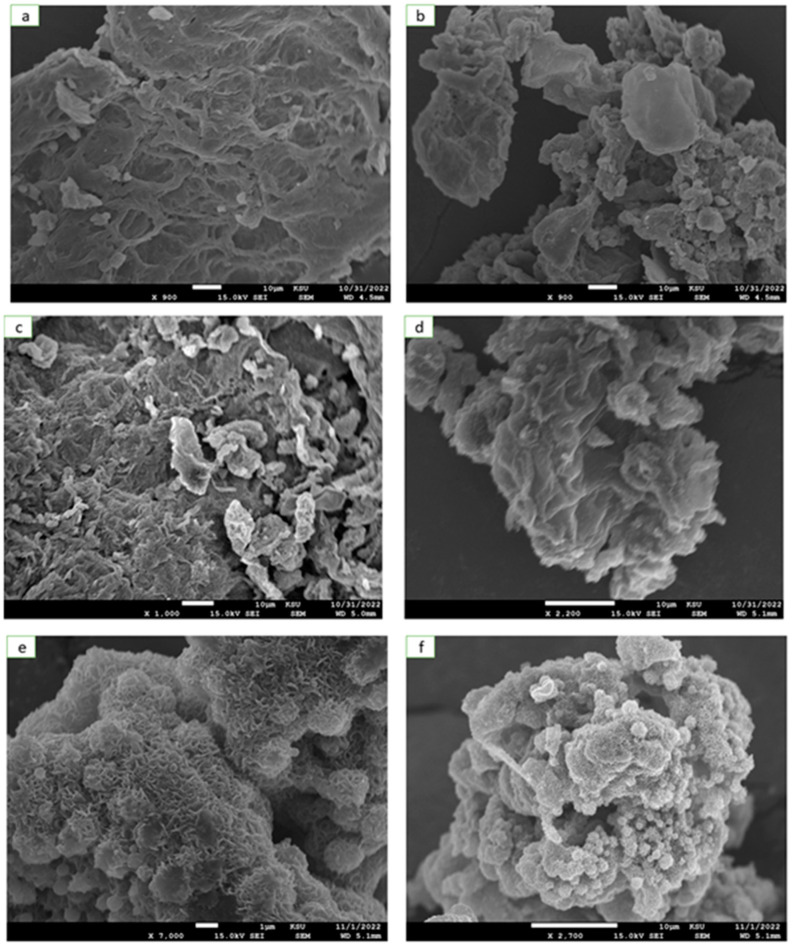
SEM images of HRG–VO_x_ nanocomposites consisting of different shapes of VO_x_ nanoparticles: HRG with VO_x_ nanorods (**a**,**b**), HRG with VO_x_ nanosheets (**c**,**d**) and HRG with VO_x_ nano-urchins (**e**,**f**).

**Figure 3:**
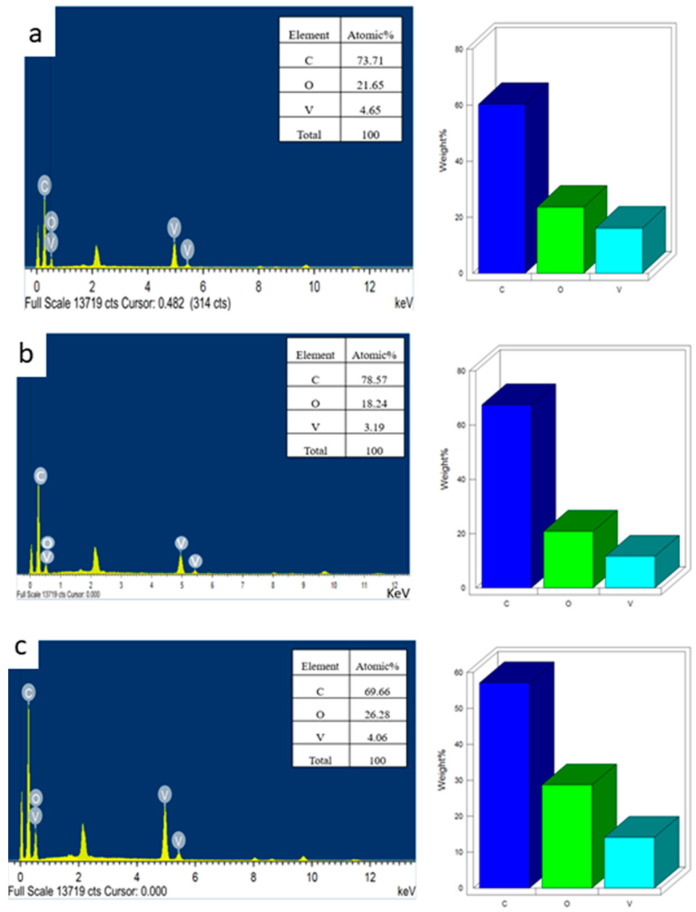
EDAX spectra of: (**a**) HRG with VO_x_ nanorods (HRG–VO_x_-R), (**b**) HRG with VO_x_ nanosheets (HRG–VO_x_-S) and (**c**) HRG with VO_x_ nano-urchins (HRG–VO_x_-U).

SEM analysis was used to determine the surface morphology of the synthesized HRG–VO_x_ nanocomposites consisting of diverse shapes of VO_x_ nanoparticles on the surface of HRG nanosheets. The SEM images in Figure 2 demonstrates the presence of pure HRG, which exhibited a crumpled paper sheet-like morphology and ultimately confirmed the layered structure of the prepared material. These layers are often folded or continuous; despite this, the edges of individual sheets can be easily distinguished, with the exception of the kinked and wrinkled areas of the HRG sheets. Figure 2a,b demonstrate the SEM images of HRG–VO_x_ nanocomposites, consisting of VO_x_ nanorods, where the layered structure of HRG is visible but the VO_x_ rods are concealed underneath the layers and thus are not visible in the SEM image of the composite. On the other hand, the HRG with VO_x_ nanosheets in Figure 2c,d show a layered structure with very fine wrinkles that are clearly visible, revealing the flower-like morphology of the composite, which can be attributed to the combination of two different types of layered materials. However, in case of HRG with the VO_x_ urchin nanocomposite, as shown in Figure 2e,f, a very fine porous structure is visible, which is very similar in morphology to the shape of a cauliflower. Ultrafine wrinkles in the SEM image of this composite can be attributed to the spikes of the urchin-shaped VO_x_ nanoparticles. In addition, the EDAX analysis also confirmed the presence of different elements, including C, O and V in the HRG–VO_x_ nanocomposites with different shapes of VO_x_ nanoparticles, as shown in Figure 3a–c. The presence of these three elements in the all the EDAX spectra of the composites clearly points towards the formation of HRG and VO_x_ composites.

**Figure 4:**
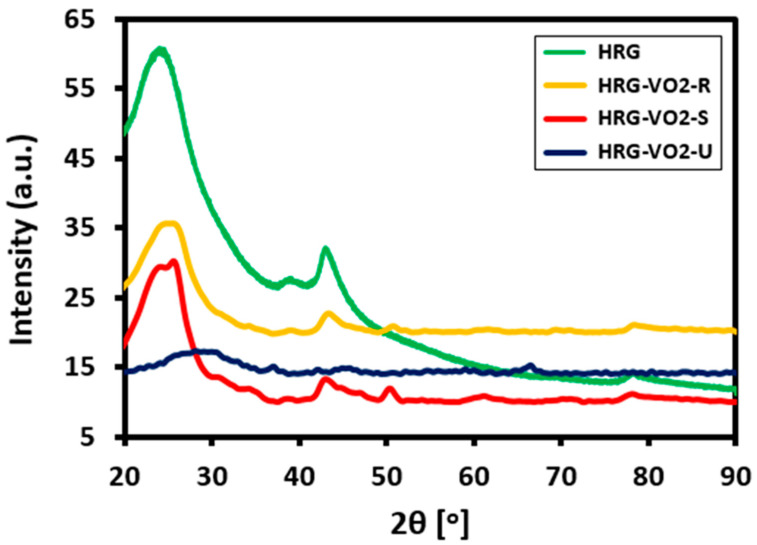
XRD patterns of pure HRG (green line), HRG with VO_x_ nanorods (yellow line), nanosheets (red line) and nano-urchins (blue line).

Apart from this, the samples were also subjected to XRD analyses to determine the crystallinity and phase compositions of the materials. Figure 4 shows the XRD patterns of pristine HRG and different HRG–VO_x_ nanocomposites. The pattern for HRG typically demonstrates two prominent peaks (Figure 4, green line) at 2θ = ~24.5° and ~42.8°, which closely matched with JCPDS No: 75-1621, corresponding to the hexagonal C (graphite) phases and representing the lattice planes (002) and (100), respectively [39]. The broad and low intensity peaks in the diffraction patterns of HRG are due to the poor crystalline natures of the same when compared to highly crystalline graphite. In all the samples, the small peak at ~43° that corresponds to the (100) reference plane can be attributed to a turbostratic disorder of carbon materials [40]. Figure 4 exhibits the X-ray diffraction patterns of the HRG–VO_x_ nanocomposites with different shapes of VO_x_ nanoparticles. X-ray diffraction patterns of the products prepared with different water:ethanol ratios and pure ethanol exhibit the reflections of VO_x_ (Figure 4, yellow, red and blue lines). Notably, due to the broad and intense peak of HRG, the characteristic peaks of VO_x_ are concealed in the baseline of the spectra. Moreover, some reflections are relatively sharper than others, which could correspond to anisotropic crystallite sizes, since according to Scherer’s formula, upon increasing crystallite size, the reflections tends to become sharper [41]. Anisotropic crystallite sizes are in good agreement with the rod-shaped morphology of the nanoparticles, as depicted in Figure 4 (yellow line) where the reflections along the length of the rods are expected to be sharper. Notably, pristine well-defined VO_x_ (B) nanorods have distinct XRD reflections, which clearly define the phase of the nanoparticles; however, in this case, the XRD patterns of the nanorods do not exactly match with the reported literature [42]. This can be attributed to the presence of vanadium in different oxidation states. For the product with pure ethanol, i.e., the urchin-shaped product (Figure 4, blue line), the profiles of the X-ray diffraction patterns are distinctly broader and less pronounced than for other shapes of VO_x_. However, most of the peaks appear almost at the same 2θ value as the other samples, which suggests that the product is indeed VO_X_ as well, albeit with very poor crystallinity.

The broad reflections, however, fit the morphology observed in the TEM images, as shown in Figure 4 (yellow, red and blue lines). It is typical for layered structures to have very broad reflections that merge into each other, making it hard to identify individual reflections. In addition, the overall crystallinity of the samples is weak leading to a high background. Therefore, it is not possible to assign the crystalline phase of the products due to the poor crystallinity of the sample and layered morphology. Hence, the results suggest that the change of the underlying motif in morphology from rods to layered structures observed using TEM is accompanied by different crystalline phases from the VO_x_ for the rods to change to a presumably layered structure.

**Figure 5:**
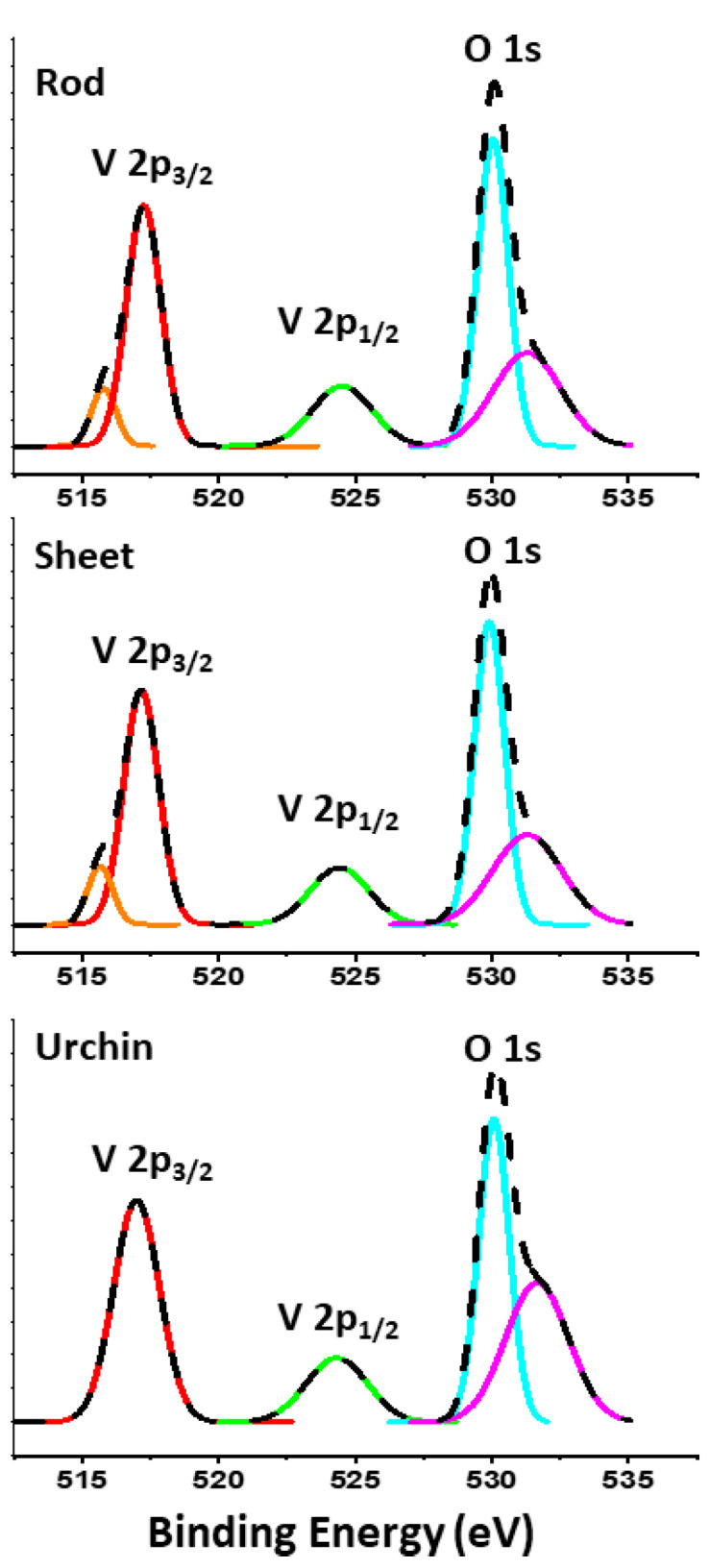
XPS analysis of HRG with VO_x_ nanorods, nanosheets and nano-urchins.

To confirm the oxidation state of vanadium in the samples, XPS analysis was performed. The XPS spectra of the as-prepared samples, as shown in Figure 5, revealed the presence of the elemental composition of V, O and C. The comparison of spectral data obtained revealed that there is a difference in the oxidation states of V obtained based on the shape. The region between 512 and 527 eV signifies the presence of V 2p, with a spin–orbit split for V 2p_3/2_ peaks in the region of 515–520 eV, while the V 2p_1/2_ peaks’ spin–orbit split falls within the range of 522–527 eV, which denotes the oxidation state of V in the nanocomposite. The rod-shaped (HRG–VO_x_-R) and sheet-shaped (HRG–VO_x_-S) morphology-based VO_x_ are mostly composed of V 3^+^, with traces of V 4^+^, while the urchin-shaped (HRG–VO_x_-U) VO_x_ is mostly V^3+^ [43]. The signal signifying the V–O bond falls in the O 1s region with a peak at 530 eV (±0.2 eV) in the case of all the three morphologies, which corresponds to vanadium oxide lattice oxygen [21,44,45]. The additional signal in the O 1s region at 531 eV corresponds to the C-O, which is due to the presence of certain oxygen moieties on the HRG surface [46]. These results further confirm that samples contain diverse shapes with mixed phases of vanadium oxide NPs on the surface of the HRG.

**Figure 6:**
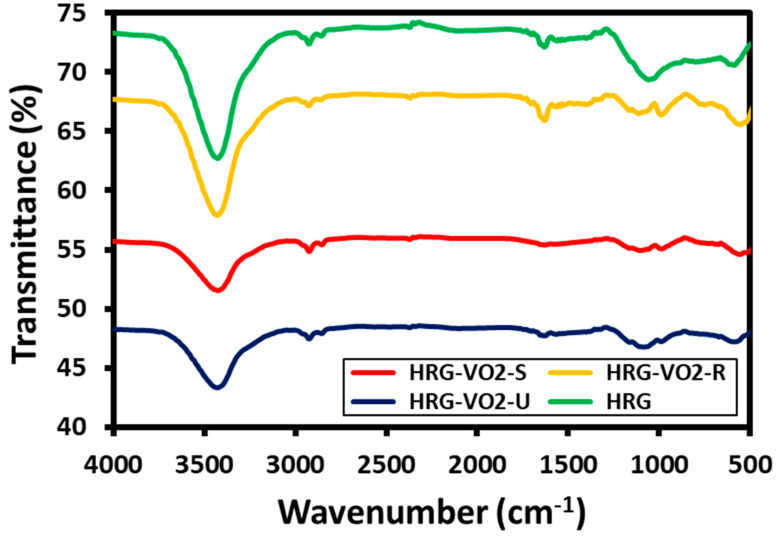
FT-IR spectra of HRG (green line), HRG with VO_x_ nanorods (yellow line), nanosheets (red line) and nano-urchins (blue line).

The FT-IR spectra of HRG in Figure 6 (green line) shows three peaks, of which the broad and intense peak at ~3440 cm^−1^ arises from the O-H stretching vibration and the peak at ~1634 cm^−1^ is due to the stretching vibrations of the aromatic C=C peak and C-OH band within the range of 1214 cm^−1^. All these peaks point towards the presence of different oxygen-containing groups on the surface of HRG. On the other hand, Figure 6 also depicts the FT-IR spectra of the HRG–VO_x_ nanorods (yellow line), HRG–VO_x_ nanosheets (red line) and HRG–VO_x_ urchins (blue line). The IR peaks in all the spectra are present in almost the same positions but with relatively different intensities. These spectra exhibit strong edge-sharing V-O stretching vibrations at ~548 cm^−^^1^ and weaker V-O-V bending vibrations at ~924 cm^−^^1^. Notably, a slight shift was observed in some of the peaks in the spectra, such as in case of urchins and sheets, which exhibited almost similar IR spectra, and the edge-sharing V-O stretching vibrations appeared at 516 cm^−^^1^ and 524 cm^−^^1^, respectively. In addition, the bands at 1050 cm^−^^1^ in all the spectra are probably due to the V=O stretching vibrations of VO_x_ nanostructures. Although the exact structures of the urchins and sheets are unknown, it can be ruled out that the IR spectra of the urchins as well as those of the sheets resemble those of the precursor VO(acac)_2_. Both spectra lack the strongest bands of VO(acac)_2_ at 1533 cm^−^^1^ and 1560 cm^−^^1^, which can be assigned to the C=C and C=O stretching bands. Thus, due to the presence of the mixed peaks of VO_x_ and HRG in the FT-IR spectra, it can be concluded that the prepared material comprises HRG–VO_x_ nanocomposites.

### Photocatalytic Degradation

**Figure 7:**
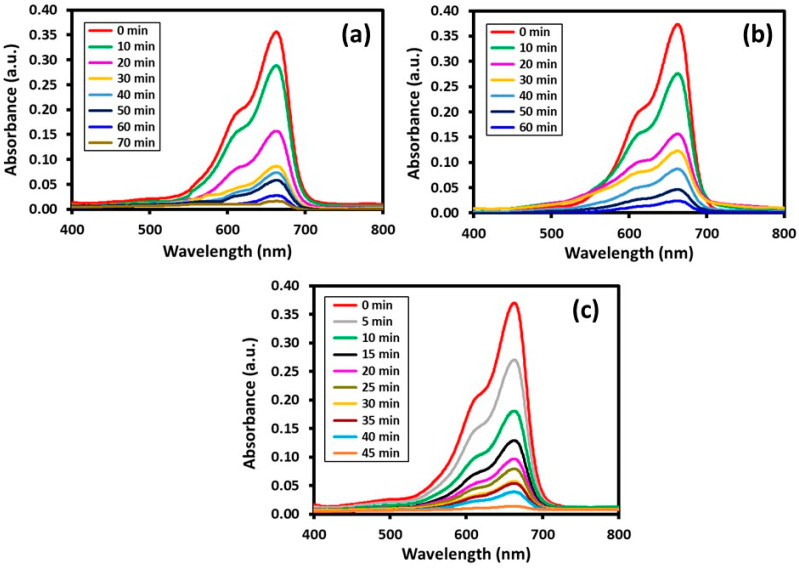
Photodegradation of MB dye using different photocatalysts, such as (**a**) HRG–VO_x_-R, (**b**) HRG–VO_x_-S and (**c**) HRG–VO_x_-U.

**Figure 8:**
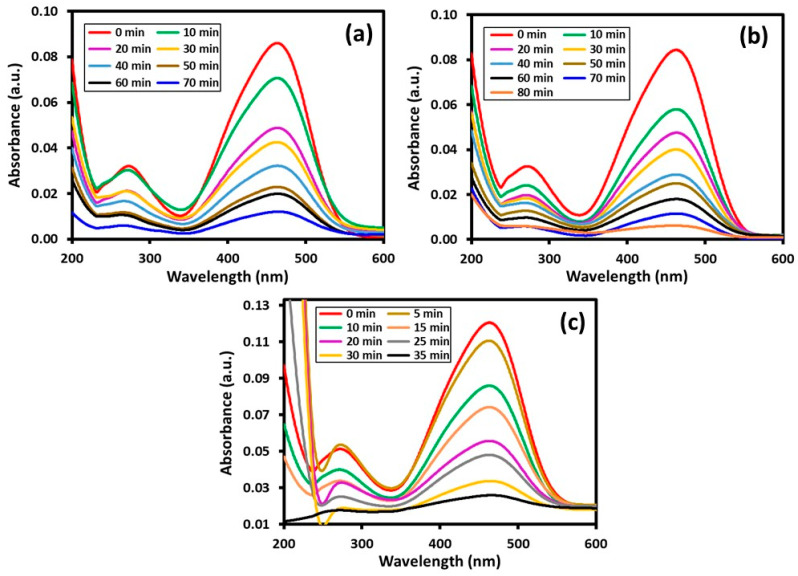
Photodegradation of MO dye: (**a**) HRG–VO_x_-R; (**b**) HRG–VO_x_-S; (**c**) HRG–VO_x_-U.

Typically, the catalytic properties of semiconductor-based photocatalysts are often dependent on several factors, including surface area, band gap, particles size, crystallinity and concentration of OH ions present on the surface of the photocatalysts. Apart from this, the morphology of the nanoparticles involved in the photocatalysts also play a crucial role in determining the photocatalytic properties of the catalyst [47]. In this study, three different photocatalysts were prepared by depositing different shaped VO_x_ nanoparticles on the surface of HRG. The results obtained are displayed in Figure 7 and Figure 8 and are also elaborated upon in Table 1. All the three samples displayed decent catalytic activity towards both the dyes, including MO and MB. Under normal conditions, for both the samples, significant degradation was observed in a short period of time. However, among these samples, HRG–VO_x_-U displayed the best photocatalytic activity in terms of the degradation of both MB and MO. As a photocatalyst, HRG–VO_x_-U degraded ~99% of MB in 45 min, while MO required only 35 min for a maximum degradation of up to ~97%. However, when HRG–VO_x_-R was used, ~70 min were required for the degradation of ~98% MB, while within the same period of time, only 86% of MO was degraded. Similarly, in the case of HRG–VO_x_-S, ~94% of MB was degraded in 60 min. Notably, HRG–VO_x_-S demonstrated relatively weak photocatalytic activity towards MO when compared to its other two counterparts, and it took a longer time (80 min) to degrade ~93% of MO. In all the cases, the final time of the reaction was decided based on the prolonged experiments performed, i.e., in each case, the reaction was allowed to continue for long time and the sample was collected at regular intervals, and the reaction was halted once no more changes were observed in the absorbance of the sample.

All the photocatalysts, including HRG–VO_x_-R, HRG–VO_x_-S and HRG–VO_x_-U displayed more or less similar photocatalytic activities. But of the three different morphologies, HRG–VO_x_-U with the urchin-shaped VO_x_ photocatalyst displayed the best photocatalytic properties, demonstrating the highest percentage of degradation in the least amount of time (cf. Table 1). The relatively high photocatalytic efficiency of HRG–VO_x_-U can be attributed to the peculiar morphology of VO_x_ nanoparticles on the surface of HRG. VO_x_ urchin nanocomposites exhibit very fine porous structures, which closely resemble the shape of a cauliflower. In addition, the composite also consists of ultrafine wrinkles, which can be attributed to the spikes of the urchin-shaped VO_x_ nanoparticles. Therefore, these pores and wrinkles may offer additional defects on the surface of the photocatalyst, which provide active sites for the absorption of substrates, thus leading to the relatively high photocatalytic properties of the catalyst. Indeed, this was further confirmed by measuring the specific surface area of the as-prepared HRG–VO_x_-R, HRG–VO_x_-S and HRG–VO_x_-U. The samples were measured via BET nitrogen adsorption after degassing at 120 °C for 16 h. It was revealed that, of all the samples, the HRG–VO_x_-U displayed a larger surface area of 42.9 m^2^ g^−1^ when compared to the surface area of HRG–VO_x_-R (23.9 m^2^ g^−1^) and HRG–VO_x_-S (32.8 m^2^ g^−1^). Notably, the enhanced surface area of HRG–VO_x_-U is attributed to the typical urchin morphology of VO_x_ NPs with spikes. Indeed, the photocatalytic properties of HRG–VO_x_-U are comparatively better than the properties of several other transitional metal oxides reported in the literature (cf. Table 2).

**Figure 9:**
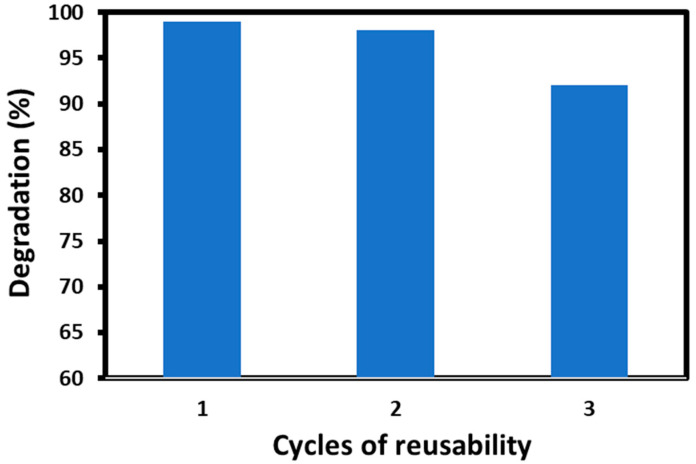
Reusability analysis of HRG–VO_x_-U for MB dye degradation under UV light.

In order to inspect reusability, the most active photocatalyst, i.e., HRG–VO_x_-U, was selected. To perform reusability tests, each time the sample was collected, it was centrifuged and recovered via filtration from the reaction mixture. Subsequently, the sample was washed a few times with DI water to remove the adsorbed impurities and was dried in oven at 60 °C for 30 min. Later, the recovered photocatalyst was reused for further reactions, and up to three cycles were repeated using a similar method. Even after three cycles, the photocatalytic activity of the sample almost remained intact, while only a slight decrease in the catalytic activity was observed after the third cycle. In the first cycle, during 45 min, HRG–VO_x_-U degraded up to ~99% of MB, while in the second cycle, the efficiency of the photocatalysts was slightly decreased and showed a degradation percentage of ~98%. On the other hand, after the third cycle, a significant decrease in photocatalytic activity was observed, where the photodegradation efficiency reached ~92% with MB. Therefore, HRG–VO_x_-U shows excellent reusability in the degradation of various dyes (Figure 9).

So far, various possible mechanisms have been proposed by researchers for the photocatalytic degradation of organic dyes, including MB and MO in aqueous solutions using similar photocatalysts [54,55]. Generally, photocatalytic degradation involving nanomaterials occured in three different steps: (i) the diffusion of dyes, in which dye molecules migrate from aqueous solution to the outer surface of the photocatalysts; (ii) the adsorbed dye molecules further migrated to the inner pores of photocatalysts, which is often referred to as intraparticle diffusion; and (iii), finally, the interaction of dye molecules with the internal pores, i.e., the active sites of photocatalysts, occurred. Similarly, in this case, in the presence of dye (MB/MO), under visible light irradiation, the HRG–VO_x_-U photocatalysts possibly absorbs the light, which leads to the promotion of electrons from the valence band (VB) to the conduction band (CB) across the band gap. Subsequently, electron–hole pairs (e−/h+) pairs are generated at the surface of the HRG–VO_x_-U. The electron at the conduction band (CB) interacts with O_2_ molecules and forms a superoxide radical, which further interacts with the H_2_O_2_ to produce the hydroxyl radical. On the other hand, the hole in the valance band (VB) interacts with water molecules (H_2_O) and produces hydroxyl radicals (^•^OH). These hydroxy radicals are highly reactive and are considered strong oxidizing agents, which facilitate the degradation of dyes into less hazardous objects and minerals [56].

## 4. Conclusions

To conclude, a facile hydro-/solvothermal preparation method was applied to obtain highly reduced graphene oxide (HRG) and vanadium oxide nanoparticle-based nanocomposites. Using this approach, different shaped VO_x_ nanoparticles, including rod-, sheet- and urchin-shaped, were successfully deposited on the surface of HRG. The compositions and morphologies of VO_x_ nanoparticles on HRG were controlled by optimizing the water:ethanol ratio. At high water concentrations in the mixture, i.e., at a water:ethanol ratio of 4:1, highly dispersed VO_x_ nanorods were obtained. In contrast, when a water:ethanol ratio of 1:9 was employed, VO_x_ nanosheets evolved, while the use of pure ethanol generated VO_x_ nano-urchins, which can be best described as congregated VO_x_ sheets. XPS revealed the formation of vanadium oxide nanoparticles (VO_x_), where the vanadium is present in mixed oxidation states, including V^+4^ and V^+6^, which was also confirmed through XRD analysis. Similarly, the presence of different shapes of VO_x_ NPs on the surface of HRG was also confirmed in SEM and TEM results. The as-prepared nanocomposites were applied in order to evaluate the shape-dependent photocatalytic properties in terms of the photocatalytic degradation of hazardous organic dyes, such as methylene blue and methyl orange. All shapes of VO_x_ NPs on the surface of HRG successfully degraded the dyes. However, of the three different shapes, the HRG–VO_x_-U nanocomposite consisting of urchin-shaped VO_x_ NPs exhibited superior photocatalytic activities.

## Figures and Tables

**Table 1:** Comparison of HRG–VO_x_ nanocomposites and their efficiencies.

			Percentage of Degradation	
Catalyst	MB	MO	MB	MO
HRG–VO_x_-R	70 min	70 min	97.9%	86.0%
HRG–VO_x_-S	60 min	80 min	93.5%	92.8%
HRG–VO_x_-U	45 min	35 min	99.1%	97.2%

**Table 2:** Comparative photocatalytic properties of various transitional metal oxides reported in the literature.

			Percentage of Degradation		References
Type of Catalyst	MB	MO	MB	MO	
ZnO NPs	35 min	35 min	~60.0%	~98.0%	[48]
ZnO NPs	60 min	60 min	~4.3%	~4.7%	[49]
Al–F∕TiO_2_ NPs	120 min	120 min	~61.0%	~83.0%	[50]
Graphene–TiO_2_ (P25)	60 min	nil	65.0%	nil	[51]
Graphene–TiO_2_ (mixed phase)	60 min	nil	97.5%	nil	[52]
V_2_O_5_	360 min	nil	60.3%	nil	[53]
HRG–VO_x_-U	45 min	35 min	99.1%	97.2%	In this study

## Data Availability

All data are contained within the article.
